# Identification of infectious disease-associated host genes using machine learning techniques

**DOI:** 10.1186/s12859-019-3317-0

**Published:** 2019-12-27

**Authors:** Ranjan Kumar Barman, Anirban Mukhopadhyay, Ujjwal Maulik, Santasabuj Das

**Affiliations:** 1 Biomedical Informatics Centre, ICMR-National Institute of Cholera and Enteric Diseases, Kolkata, West Bengal India; 2 Department of Computer Science and Engineering, Jadavpur University, Kolkata, West Bengal India; 3 Department of Computer Science and Engineering, University of Kalyani, Kalyani, West Bengal India; 4 Division of Clinical Medicine, ICMR-National Institute of Cholera and Enteric Diseases, P-33, C.I.T.Road Scheme XM, Beliaghata-700010, Kolkata, West Bengal India

**Keywords:** Classification, Deep neural networks, Functional annotations, Infectious disease-associated host genes, Sequence and interaction network features

## Abstract

**Background:**

With the global spread of multidrug resistance in pathogenic microbes, infectious diseases emerge as a key public health concern of the recent time. Identification of host genes associated with infectious diseases will improve our understanding about the mechanisms behind their development and help to identify novel therapeutic targets.

**Results:**

We developed a machine learning techniques-based classification approach to identify infectious disease-associated host genes by integrating sequence and protein interaction network features. Among different methods, Deep Neural Networks (DNN) model with 16 selected features for pseudo-amino acid composition (PAAC) and network properties achieved the highest accuracy of 86.33% with sensitivity of 85.61% and specificity of 86.57%. The DNN classifier also attained an accuracy of 83.33% on a blind dataset and a sensitivity of 83.1% on an independent dataset. Furthermore, to predict unknown infectious disease-associated host genes, we applied the proposed DNN model to all reviewed proteins from the database. Seventy-six out of 100 highly-predicted infectious disease-associated genes from our study were also found in experimentally-verified human-pathogen protein-protein interactions (PPIs). Finally, we validated the highly-predicted infectious disease-associated genes by disease and gene ontology enrichment analysis and found that many of them are shared by one or more of the other diseases, such as cancer, metabolic and immune related diseases.

**Conclusions:**

To the best of our knowledge, this is the first computational method to identify infectious disease-associated host genes. The proposed method will help large-scale prediction of host genes associated with infectious-diseases. However, our results indicated that for small datasets, advanced DNN-based method does not offer significant advantage over the simpler supervised machine learning techniques, such as Support Vector Machine (SVM) or Random Forest (RF) for the prediction of infectious disease-associated host genes. Significant overlap of infectious disease with cancer and metabolic disease on disease and gene ontology enrichment analysis suggests that these diseases perturb the functions of the same cellular signaling pathways and may be treated by drugs that tend to reverse these perturbations. Moreover, identification of novel candidate genes associated with infectious diseases would help us to explain disease pathogenesis further and develop novel therapeutics.

## Background

Infectious diseases are continue to be a major threat to public health, regardless of the recent advances in sanitation, immunization, and antimicrobial therapy. According to a report from World Health Organization (WHO), infectious diseases remain a concern to all countries, resulting in a sizeable number of deaths and imposing a significant burden on the economy [1]. Especially, in the low income and low-middle income countries, infectious diseases are the leading cause of mortality for children. Infectious diseases are caused by a wide variety of pathogenic microorganisms, including viruses, bacteria, protozoa and fungi. The outcome of the host-pathogen interactions is either the development of the disease or clearing of the organism by the host immune system. For disease development, pathogens influence critical biological processes in the host cells to escape the immune system [2]. Identification of the regulation of host genes by pathogens is critical for better understanding of the mechanisms underlying the development of infectious diseases.

Majority of the disease-related studies conducted so far focused primarily on the single nucleotide polymorphisms (SNPs) [3–6]. Attempts have been made of late to integrate the information on disease-associated genes available at different public repositories [7, 8]. The recent spurt of knowledge on genomics has boosted bioinformatics research to computationally predict as well as prioritize disease-associated genes. Although several methods have been proposed for gene prioritization [9–16], majority are related to Mendelian diseases and few others to complex diseases like asthma, diabetes and cancer. These methods have utilized various biological information, such as gene co-expression, gene ontology (GO) annotation, protein-protein interaction (PPI) networks, domain, motif and sequence information etc. In addition, machine learning approaches using protein-protein interaction network properties, sequence and functional features were applied to identify cancer and Alzheimer disease-associated genes [17, 18]. However, no methods have been developed so far to predict the host genes associated with infectious diseases.

We have used machine learning techniques (MLT) and employed sequence and protein-protein interaction network properties to predict infectious disease-associated host genes. Deep Neural Networks (DNN) methods were shown to perform well with a number of diverse problems. Since, DNN is becoming a popular algorithm in the field of modern computer science, we primarily focused on DNN. However, the performance of DNN model was also compared with other well-known classifiers, such as Support Vector Machine (SVM), Naïve Bayes (NB) and Random Forest (RF). We validated the performance of our model on both blind (not used in training or testing) and independent datasets. In addition, to identify novel genes, we applied the model to all reviewed proteins, which were not used as the blind dataset or for the training or testing purposes. Finally, highly predicted proteins were studied for host-pathogen PPIs and validated by functional annotation, including disease and gene ontology enrichment analysis.

## Results

### Selection of features

We tested different combinations of primary sequence features and topological (network) features to attain a high level of accuracy, sensitivity and specificity. As shown in Table 1 (Complete information available in Additional file 1: Table S16), network properties features (9) were able to achieve an accuracy of 84.43%, with sensitivity and specificity approaching 78.24% and 90.51%, respectively. Furthermore, we observed that normalized and filtered network properties features (6 features) achieved the best accuracy (84.76%), with sensitivity of 77.77% and specificity of 91.71%. Among the primary sequence features, AAC, PAAC and combination of both were found to perform marginally better than the other features.

**Table 1:** Features wise performance measures on disease and non-disease associated proteins dataset using deep neural network classifier

Primary sequence features									
Features set	Vector length	*P*(+): *N*(−)	Sensitivity (%)	Specificity (%)	Accuracy (%)	PPV (%)	MCC	F1 score (%)	AUC
AAC	20	1: 1	86.32	53.31	70.09	66.04	0.43	74.34	0.755
PAAC	50	1: 1	86.32	53.31	70.09	66.04	0.43	74.34	0.755
CTD	343	1: 1	91.09	37.87	64.52	59.52	0.34	71.86	0.692
DC	400	1: 1	88.59	44.63	66.83	62.96	0.38	72.89	0.715
AAC_PAAC	70	1: 1	85.15	59.93	72.98	69.02	0.47	75.92	0.766
AAC_CTD	363	1: 1	87.45	47.18	67.74	62.83	0.39	72.81	0.709
AAC_DC	420	1: 1	83.55	52.72	68.73	64.66	0.39	72.69	0.708
PAAC_CTD	393	1: 1	88.52	45.23	67.02	62.46	0.39	72.78	0.720
PAAC_DC	450	1: 1	88.08	50.40	69.73	65.24	0.43	74.40	0.732
CTD_DC	743	1: 1	87.15	48.30	67.94	64.59	0.40	73.08	0.733
AAC_PAAC_CTD	413	1: 1	83.72	53.77	68.96	64.93	0.40	72.72	0.730
AAC_PAAC_DC	470	1: 1	86.32	52.49	69.86	65.64	0.43	74.09	0.729
AAC_CTD_DC	763	1: 1	90.22	45.17	67.88	62.69	0.40	73.72	0.729
PAAC_CTD_DC	793	1: 1	90.30	45.27	67.80	63.62	0.40	73.94	0.743
AAC_PAAC_CTD_DC	813	1: 1	87.50	49.44	68.50	64.00	0.41	73.50	0.739
Network Analyzer properties									
Network properties	**9**	1: 1	**78.24**	**90.51**	**84.43**	**89.22**	**0.69**	**83.24**	**0.858**
Normalized And Filtered Network properties	**6**	1: 1	**77.77**	**91.71**	**84.76**	**90.45**	**0.70**	**83.44**	**0.856**

The notable performances are indicated by bold

To accomplish nearly equivalent sensitivity and specificity along with high accuracy, we also tested different combinations of AAC, PAAC and network properties features. As shown in Table 2 (Complete information available in Additional file 1: Table S17), the combination of PAAC and network properties features (59) achieved the best accuracy (86.94%) along with high sensitivity (86%) and specificity (87.48%).

**Table 2:** Mixed features based performance on disease and non-disease associated proteins dataset

Mixed features										
Features set	Methods	Vector length	*P*(+): *N*(−)	Sensitivity (%)	Specificity (%)	Accuracy (%)	PPV (%)	MCC	F1 score (%)	AUC
AAC_Network properties	DNN	29	1: 1	82.23	88.30	85.41	88.10	0.71	84.91	0.900
PAAC_Network properties	DNN	**59**	**1**: **1**	**86.00**	**87.48**	**86.94**	**87.93**	**0.74**	**86.76**	**0.909**
AAC_PAAC_ Network properties	DNN	**79**	**1**: **1**	**86.81**	**85.27**	**86.12**	**85.89**	**0.72**	**86.25**	**0.905**
Normalized And Filtered AAC_Network properties	DNN	26	1: 1	83.78	86.90	85.51	86.95	0.71	85.21	0.904
Normalized And Filtered PAAC_Network properties	DNN	**41**	**1**: **1**	**85.54**	**86.46**	**86.08**	**86.52**	**0.72**	**85.96**	**0.902**
Normalized And Filtered AAC_PAAC_Network properties	DNN	**60**	**1**: **1**	**85.54**	**87.36**	**86.56**	**87.68**	**0.73**	**86.45**	**0.909**

The notable performances are indicated by bold

Subsequently, we applied ensemble features selection (EFS) on the set of features, which achieved accuracies greater than 86% (Shown in Table 2 as a bold row). We found that selected features from EFS were also able to achieve similar performance levels. Finally, we identified 10 selected features (Additional file 1: Table S18) for normalized and filtered PAAC_Network properties and 16 selected features (Additional file 1: Table S19) for PAAC_Network properties, which were able to obtain accuracies of 86.44% and 86.33%, respectively (Table 3 and Complete information available in Additional file 1: Table S20). Together the above results suggested that 10 and 16 features sets achieved the highest levels of accuracy with equivalent performance.

**Table 3:** Selected features wise performance measures using different classifier

Features set	Methods	Vector length	*P*(+): *N*(−)	Sensitivity (%)	Specificity (%)	Accuracy (%)	PPV (%)	MCC	F1 score (%)	AUC
Selected Features For PAAC_Network properties	DNN	**16**	**1**: **1**	**85.61**	**86.57**	**86.33**	**86.91**	**0.73**	**86.15**	**0.899**
Selected Features For PAAC_Network properties	SVM	16	1: 1	78.03	87.87	82.95	86.40	0.66	81.81	0.862
Selected Features For PAAC_Network properties	RF	16	1: 1	83.93	88.03	85.98	87.52	0.72	85.69	0.916
Selected Features For PAAC_Network properties	NB	16	1: 1	78.03	88.03	83.03	86.70	0.66	82.14	0.904
Selected Features For AAC_PAAC_Network properties	DNN	**24**	**1**: **1**	**84.72**	**88.08**	**86.60**	**87.97**	**0.73**	**86.18**	**0.907**
Selected Features For AAC_PAAC_Network properties	SVM	24	1: 1	80.00	87.87	83.93	86.64	0.68	83.01	0.881
Selected Features For AAC_PAAC_Network properties	RF	24	1: 1	82.62	87.70	85.16	87.05	0.70	84.78	0.918
Selected Features For AAC_PAAC_Network properties	NB	24	1: 1	78.52	88.36	83.44	87.09	0.67	82.59	0.911
Selected Features For Normalized And Filtered PAAC_Network properties	DNN	**10**	**1**: **1**	**84.62**	**87.63**	**86.44**	**88.06**	**0.73**	**86.00**	**0.894**
Selected Features For Normalized And Filtered PAAC_Network properties	SVM	10	1: 1	77.54	87.70	82.62	86.34	0.66	81.48	0.880
Selected Features For Normalized And Filtered PAAC_Network properties	RF	10	1: 1	81.15	86.39	83.77	85.64	0.68	83.33	0.910
Selected Features For Normalized And Filtered PAAC_Network properties	NB	10	1: 1	76.23	91.31	83.77	89.77	0.68	82.45	0.896
Selected Features For Normalized And Filtered AAC_PAAC_Network properties	DNN	**25**	**1**: **1**	**87.03**	**85.07**	**86.45**	**86.77**	**0.73**	**86.66**	**0.908**
Selected Features For Normalized And Filtered AAC_PAAC_Network properties	SVM	25	1: 1	78.85	88.52	83.69	87.07	0.68	82.56	0.889
Selected Features For Normalized And Filtered AAC_PAAC_Network properties	RF	25	1: 1	81.64	86.72	84.18	86.01	0.68	83.77	0.911
Selected Features For Normalized And Filtered AAC_PAAC_Network properties	NB	25	1: 1	77.38	89.67	83.52	88.22	0.68	82.45	0.908

The notable performances are indicated by bold

### Performance comparison of different classifiers

**Fig. 1:**
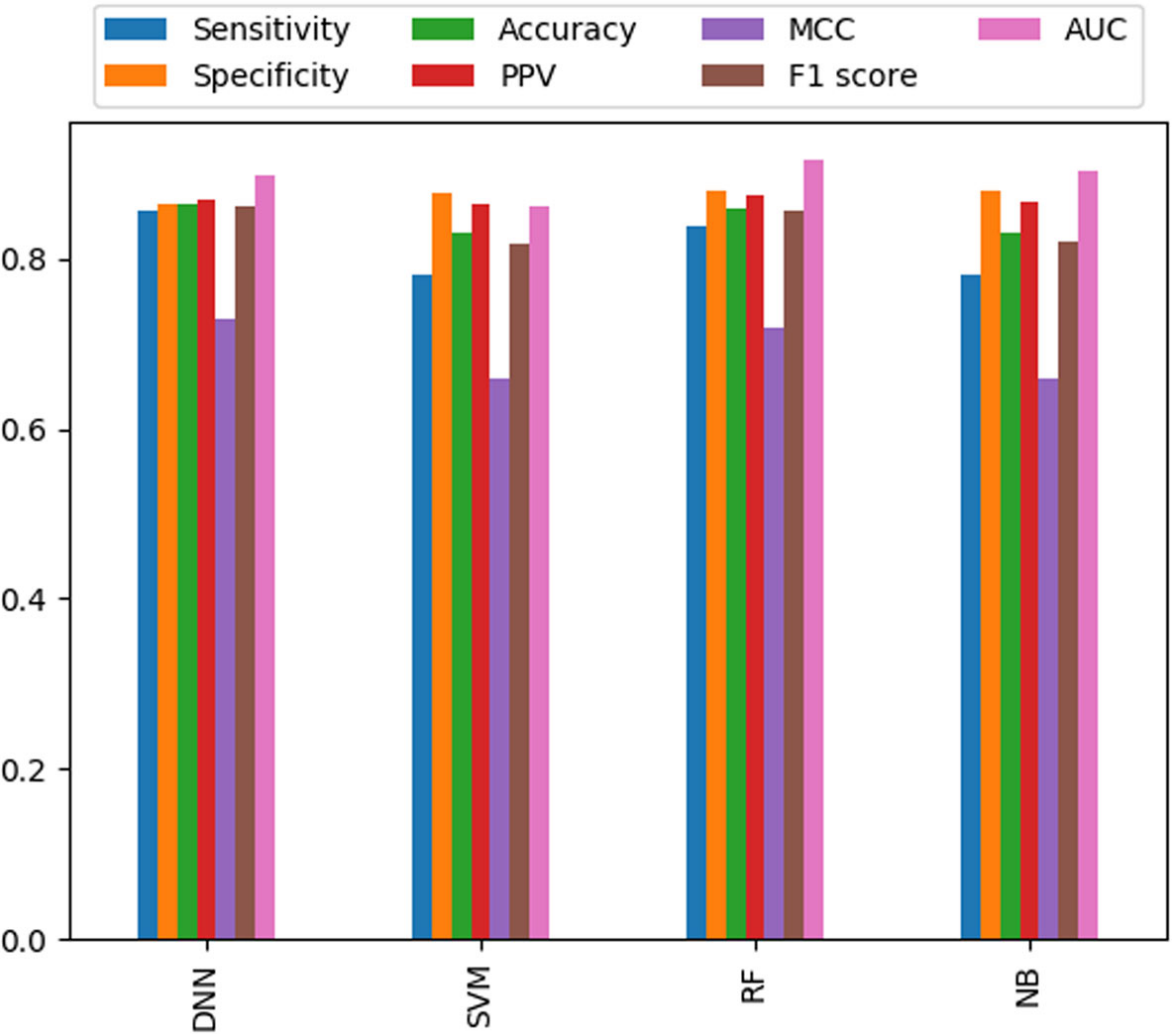
Performance measures of different classifiers based on 16 selected features from pseudo-amino acid composition (PAAC) and network properties

The performance measures of different classifiers, such as SVM, NB and RF for our dataset were compared with the DNN classifiers. We calculated the performance based on different parameters and reported only the best results for each classifier. As shown in Table 3 and Fig. 1 (Complete information available in Additional file 1: Table S20), DNN and RF performed better than SVM and NB. Furthermore, we found that the performance of DNN was more balanced and marginally better than RF.

### Performance on imbalanced datasets

In order to closely resemble real-world situations, where size of the negative dataset is much larger than the positive dataset, we tested the performance of our model on imbalanced datasets. We observed that the specificity gradually increased with an increase in the size of the negative dataset (Table 4). As a result, overall accuracy also increased. However, sensitivity decreased when the size of the negative dataset increased. In case of imbalanced datasets, accuracy is not a perfect performance measure for classification. Instead, MCC, F1-score and AUC are better performance measures for the classification of such datasets [19]. As shown in Table 4, performance was better when the positive and negative datasets were of equal size (balanced) compared with the imbalanced datasets.

**Table 4:** Performance on imbalanced datasets using deep neural network classifier

Features set	Vector length	*P*(+): *N*(−)	Sensitivity (%)	Specificity (%)	Accuracy (%)	PPV (%)	MCC	F1 score (%)	AUC
Selected Features For PAAC _Network properties	**16**	**1**: **1**	**85.61**	**86.57**	**86.33**	**86.91**	**0.73**	**86.15**	**0.899**
Selected Features For PAAC _Network properties	16	1: 2	77.89	92.56	87.81	84.64	0.72	80.72	0.900
Selected Features For PAAC _Network properties	16	1: 3	72.34	94.54	89.03	81.70	0.70	76.53	0.902
Selected Features For PAAC _Network properties	16	1: 4	68.89	95.46	90.20	79.20	0.68	73.52	0.897
Selected Features For PAAC _Network properties	16	1: 5	69.00	95.13	90.85	74.44	0.66	71.25	0.895
Selected Features For Normalized And Filtered PAAC_ Network properties	**10**	**1**: **1**	**84.62**	**87.63**	**86.44**	**88.06**	**0.73**	**86.00**	**0.894**
Selected Features For Normalized And Filtered PAAC_ Network properties	10	1: 2	76.76	92.94	87.62	84.41	0.72	80.25	0.895
Selected Features For Normalized And FilteredPAAC_ Network properties	10	1: 3	74.35	93.52	88.91	80.40	0.70	76.88	0.895
Selected Features For Normalized And Filtered PAAC_ Network properties	10	1: 4	67.39	96.27	90.57	82.68	0.69	73.66	0.897
Selected Features For Normalized And Filtered PAAC_ Network properties	10	1: 5	67.52	96.01	91.31	77.95	0.67	71.97	0.895

The notable performances are indicated by bold

### Performance on blind dataset

We tested the performance of our model on blind dataset (not used in the training or testing to build the prediction model). As shown in Table 5, selected features for normalized and filtered PAAC_Network properties (10 features) and selected features for PAAC_Network properties (16 features) achieved accuracies of 84.65% and 83.33%, respectively with the blind dataset.

**Table 5:** Performance on blind dataset using best deep neural network classifier

Best Model Features set	Vector length	*P*(+): *N*(−)	Sensitivity (%)	Specificity (%)	Accuracy (%)	PPV (%)	MCC	F1 score (%)	AUC
PAAC_Network properties	59	1: 1	85.09	76.32	80.70	78.23	0.62	81.51	0.872
Selected Features For PAAC _Network properties	**16**	**1**: **1**	**89.47**	**77.19**	**83.33**	**79.69**	**0.67**	**84.30**	**0.904**
Selected Features For Normalized And Filtered PAAC_ Network properties	**10**	**1**: **1**	**88.60**	**80.70**	**84.65**	**82.11**	**0.70**	**85.23**	**0.879**

The notable performances are indicated by bold

### Performance on independent dataset

We applied two best DNN models to independent dataset for the purpose of prediction. We found that DNN models based on the selected features for normalized and filtered PAAC_Network properties (10 features) and selected features for PAAC_Network properties (16 features) predicted 88 and 118 proteins, respectively as positives out of 142 independent infectious disease-associated proteins. These models were the best and attained the sensitivity of 61.97% and 83.10%, respectively on independent datasets (Additional file 1: Table S21). Therefore, we considered the DNN with 16 selected features for PAAC_Network properties as the proposed model for the prediction of infectious disease-associated host genes.

### Functional annotation

**Fig. 2:**
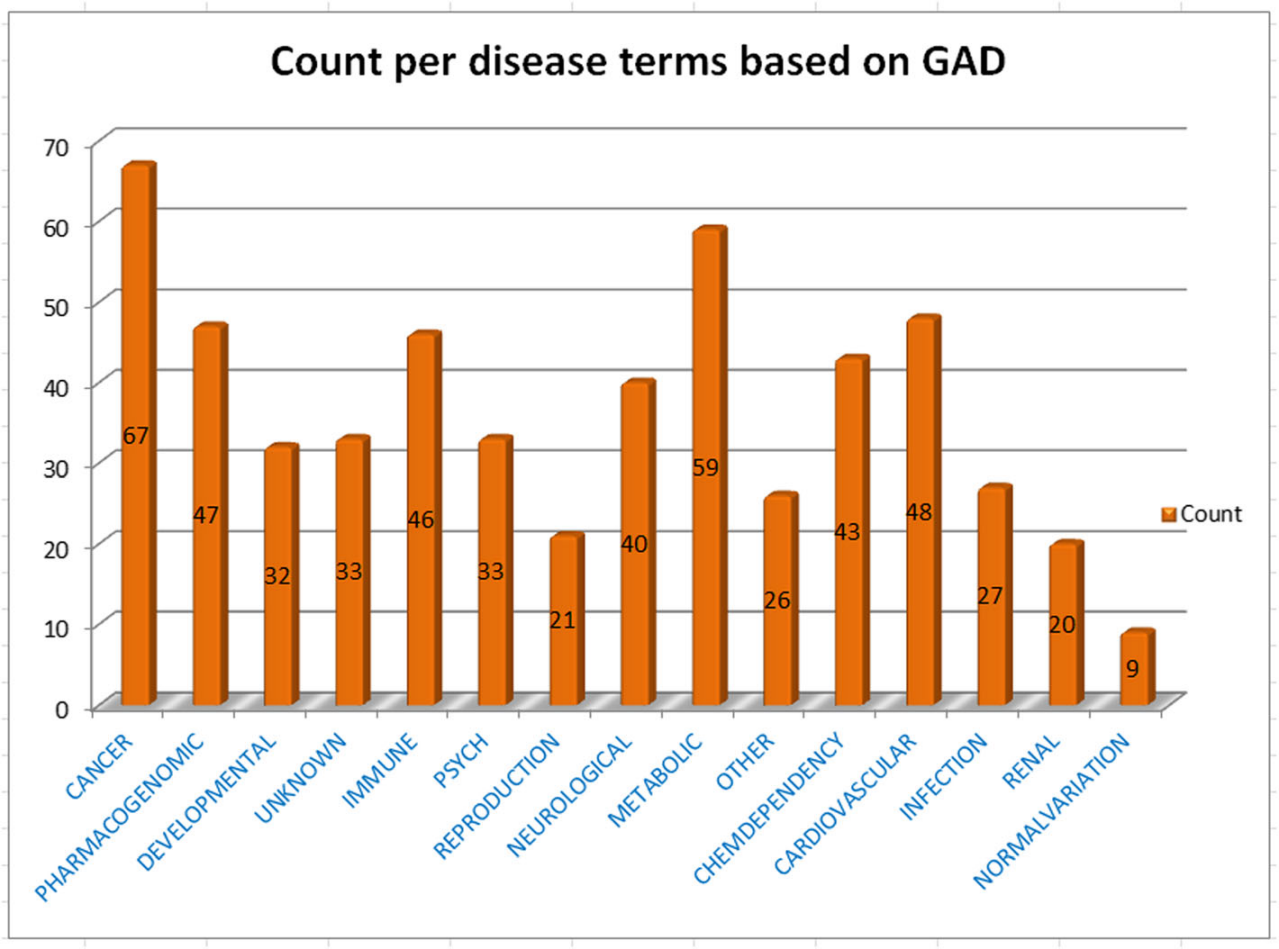
Histogram representation of different disease terms based on GAD

**Fig. 3:**
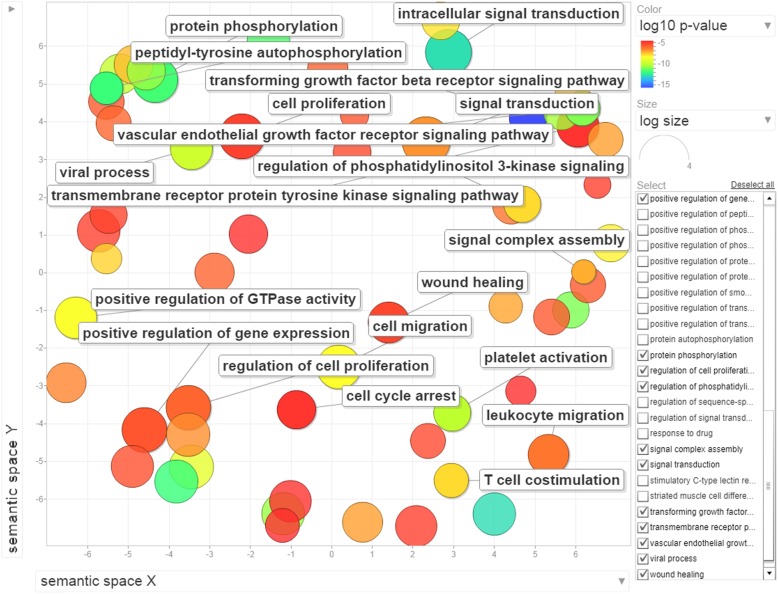
Scatter plot of significantly enriched GO biological process terms, visualized by REVIGO summarizes and visualizes long lists of gene ontology terms [21]

Finally, the set of all reviewed human proteins, not used for the training or testing purposes or as a blind dataset were predicted by our proposed model for their association with infectious diseases. Top 100 highly predicted proteins positively related to infectious diseases were considered for functional annotation (Additional file 1: Table S22). We found that 76 out of 100 highly-predicted proteins were present in the experimentally-verified host-pathogen PPIs databases, namely PHISTO [20] (Additional file 4: Fig. S3). Disease ontology enrichment analysis showed that 67, 59, 46 and 27 out of 100 proteins were classified as disease terms, viz., cancer, metabolic, immune and infection, respectively (Fig. 2 and Additional file 1: Table S23). It is noteworthy that 12 proteins out of 100 were common for cancer, metabolic, immune and infection disease terms (Additional file 5: Figure. S4). In addition, we observed that the highly-predicted infectious disease-associated proteins were also found in cancer, metabolic and immune disease terms. Gene ontology enrichment analysis showed that the genes corresponding to the above proteins were enriched in biological processes like, intracellular signal transduction (GO:0035556), protein phosphorylation (GO:0006468), signal transduction (GO:0007165), transforming growth factor beta receptor signaling pathway (GO:0007179) and viral processes (GO:0016032) (Fig. 3 and Additional file 1: Table S24).

## Discussion

Mechanisms behind the development of infectious diseases remain elusive in many cases due to the ever-changing mode of pathogen adaptation to the host systems. Identification of infectious disease-associated host genes is critical to explore the underlying mechanisms and combat infectious diseases. Although experimental techniques are best to address these problems, computational approaches promise better economy, in terms of money, time and labour. In addition, increasing availability of information in the public domain has made computational identification of disease-associated genes easier and more accurate.

Despite the existence of a large number of infectious diseases with diverse clinical and biochemical features, they have several commonalities, such as acute onset in most cases, transmissibility between the hosts, immune response patterns of the host and the response to antimicrobial agents, which prompted their classification as one broad entity. Similarly, different cancers were considered as a single entity and MLT was applied for the prediction of host genes related to cancer despite considerable variability [17]. Host response due to infection is distinct from non-infectious diseases and initiated by the engagement of microbe- or pathogen-associated molecular patterns (MAMPs or PAMPs) by the innate recognition receptors (for eg, Toll-like or NOD-like receptors). In this study, we have introduced a MLT-based computational approach to identify infectious disease-associated host genes by integrating sequence and PPI network properties features. It was earlier reported that sequence features alone were not sufficient for efficient identification of disease-related host genes. Similarly, for infectious diseases, we have observed that sequence features-based prediction models performed poorer than the models based on PPI network properties features for host gene prediction (Table 1). AAC and PAAC performed marginally better than other sequence composition features, while introduction of PPI network properties features further improved the accuracy in our study and the same was observe by other groups as well (Table 1). We found that prediction models based on the combination of sequence and network properties features achieved higher performance levels than either feature considered individually (Table 2). Based on the latest advances in the processing power and storage capacity of the computers, DNN classifier has gained popularity as it performed well for diverse data. We found that DNN classifier performed marginally better than RF, SVM and NB (Table 3). To further improve the performance of DNN classifier, we employed TensorFlow DNN, which is a widely-used deep learning package nowadays for the classification of infectious disease-associated and non-disease-associated host proteins. We have executed the whole process using the TensorFlow DNN as opposed to H2O DNN that we used earlier and found that the TensorFlow DNN method achieved a higher accuracy of ~ 96% along with the sensitivity and specificity of ~ 96% each with the training set. However, the model performance dropped significantly with the test dataset, where it achieved an accuracy of 83% with comparable sensitivity (81.2%) and specificity (85.1%). We had also applied TensorFlow DNN to another small dataset (less than 1000 positive) and found similar performance measure. Since deep learning method is specifically designed to deal with large datasets and large set of features, it performs better than RF and SVM for large datasets and large set of features. We concluded that for small datasets and small number of features set, deep learning method had marginal advantage over RF and SVM. However, this small difference may be important for the development of prediction models. Since the primary goal of our study was to design a prediction model for infectious disease-associated host genes, we searched for simple sequence and network features, which would efficiently serve this purpose. Given that a single feature selection method may have bias, we employed ensemble feature selection techniques, which achieved a performance level similar to that of the corresponding all features (Tables 2 and 3). We observed that positive and negative datasets of equal size (balanced) performed better than the imbalanced datasets and achieved nearly equal sensitivity and specificity, which is ideal for any prediction model (Table 4).

To the best of our knowledge, no computational or MLT-based method has been developed to-date to identify infectious disease-associated host genes. Therefore, we compared our method with the existing MLT-based methods, which were used for the prediction of host genes related to other diseases like cancer and Alzheimer Disease. Liu et al. achieved the highest AUC of 0.834 with the use of MLT for cancer disease-associated host gene prediction, while our method for infectious diseases achieved an AUC of 0.899. MLT applied to another study identified genes associated with Alzheimer Disease, with the maximum accuracy of 79.9%, F1-score of 15.6% and MCC of 0.201 (Jamal et al.). In contrast, our method achieved an accuracy of 86.33%, F1-score of 86.15% and MCC of 0.733 for infectious diseases. This underscores the validity of our proposed model for identifying disease-associated host genes. We found 724 infectious disease-associated host genes from 60 infectious diseases (IDs). If each of these diseases was considered a different entity, we would find less than 4 host genes for most diseases. It might be scientifically incorrect to develop any classifier using such small set of genes and not in agreement with the primary goal of this study, which was to computationally predict infectious-disease associated host genes. It is well established that host-pathogen PPIs play a major role for the pathogenesis of infectious diseases. We found that majority (76) of our highly-predicted proteins (100) were from the virus-human and bacteria-human PPIs. This indicates that our proposed model would perform well in the contexts of infectious diseases. Top 100 proteins predicted by us were further validated by disease and gene ontology enrichment analysis. The important biological processes, such as intracellular signal transduction, protein phosphorylation, signal transduction, cell proliferation, cell cycle arrest, cell migration, leukocyte migration and wound healing, which are critical events during the pathogenesis of infectious diseases, were detected by gene ontology enrichment analysis. Disease ontology enrichment analysis showed that highly predicted genes were associated with infection disease term as well as terms like cancer, metabolic, immune etc. This suggests that many critical cellular signalling pathways are common targets of the infectious and other diseases and thus, drugs used to treat other ailments may be repurposed for the host-targeting therapies of infectious diseases.

## Conclusions

Identification of genes associated with infectious diseases may help the scientific community to identify disease risks and therapeutic targets. Majority of the computational approaches available to-date are meant for the prediction of genes associated with cancer and Alzheimer disease. We propose here a computational approach for the prediction of infectious disease associated host genes. Our proposed model is based on the integration of integrating sequence and PPI network properties features. Overall, the model achieved an accuracy of 86.33%, F1-score of 86.15% and MCC of 0.733 and AUC of 0.899. The validity of our model is underscored by the identification of the genes known to be involved in important biological processes during the pathogenesis of infectious disease as the top predicted genes. Identification of novel candidates in the pool of infectious disease-associated host genes will expand our knowledge on disease pathogenesis and might help to design new therapies.

## Methods

### Collection of data

Disease-associated human genes were collected from DisGeNET [8], a database comprehensively integrated expert-curated and text-mining derived disease-associated genes from various public repositories and literatures. This database considered public repositories like GWAS Catalog [5], Comparative Toxicogenomics Database (CTD) [22], UniProtKB [23], ClinVar [24], Orphanet [25], Rat Genome Database (RGD) [26], Mouse Genome Database (MGD) [27], Genetic Association Database (GAD) [28], Literature Human Gene Derived Network (LHGDN) [29] and BeFree data [30, 31].

We have downloaded all curated gene-disease association dataset from DisGeNET and extracted only the infectious disease-associated genes (Additional file 1: Table S1). We found 745 unique human genes associated with different infectious diseases. All these gene names were mapped to Uniprot Id using mapping table of DisGeNET. 724 out of 745 gene names were mapped to Uniprot Id (Additional file 1: Table S2). Furthermore, we have found these 724 human proteins from 60 types of infectious diseases. If we considered these 60 infectious diseases as separate entities, we would find less than 4 human proteins for most of the diseases. Therefore, we considered all the above host proteins as a single group of infectious disease-associated proteins. Next, we used 610 out of 724 infectious disease-associated proteins as a positive dataset (Additional file 1: Table S3) and the remaining 114 proteins as a blind positive dataset (not used in the training or testing for building the prediction model) (Additional file 1: Table S4).

We also extracted all the disease-associated (14,623) and reviewed (20,244) human proteins from the DisGeNET and UniProtKB databases, respectively (Additional file 1: Tables S5, S6). We considered 5621 reviewed human proteins not associated with any diseases as non-disease associated proteins (Additional file 2: Figure. S1 and Additional file 1: Table S7), of which 3050 (5 times bigger than positive dataset) randomly selected proteins were treated as the negative dataset (Additional file 1: Table S8). Furthermore, we randomly selected 114 (similar size of the blind positive dataset) out of the remaining 2571 (5621–3050) non-disease associated proteins and treated them as a blind negative dataset (Additional file 1: Table S9).

For the purpose of validation, we collected Befree text mining genes from DisGeNET, which were associated with infectious diseases. Subsequently, we filtered the genes using DisGeNET confidence score greater than 0.002738764 (average DisGeNET confidence score of all Befree text mining genes associated with infectious diseases) and found 272 unique genes. We found that 128 out of 272 genes were present in our positive dataset (Additional file 3: Figure. S2). Thus, we considered only the remaining 144 (272–128) genes. Among them, 142 were mapped to Uniprot Id using the mapping table of DisGeNET. Finally, we considered these 142 proteins as the independent dataset (Additional file 1: Table S10).

### 10-fold cross-validation

We used 10-fold cross-validation techniques to elude the performance biased of all prediction methods. The entire dataset was distributed into 10 segments or folds of equal or nearly equal sizes. Training and testing were repeated 10 times with one set (fold) going out for testing, while the remaining 9 sets (folds) were used for training each time. The overall performance of the model was measured by the average performance over 10 folds.

### Features

Protein Sequence features, including amino acid composition (AAC), dipeptide composition (DC), pseudo-amino acid composition (PAAC) and conjoint triad descriptors (CTD) were used extensively in the field of computational biology [32–37]. We used AAC, DC, PAAC and CTD for the prediction of infectious disease associated human proteins. Protein sequence features were calculated using “protr” R package [38].

We retrieved expert-curated human protein-protein interactions (PPIs) from the Human Protein Reference Database (HPRD) (Release 9) to compute topological features for human proteins [39]. HPRD comprises of 39,240 binary human PPIs between 9617 proteins (Additional file 1: Tables S11, S12). Afterward, we mapped the gene name to Uniprot Id using Id mapping tool of Uniprot [23] and found 36,558 human PPIs involving 8991 proteins (Additional file 1: Tables S13, S14). The topological properties, such as average shortest path length, betweenness centrality, closeness centrality, clustering coefficient, degree, eccentricity, neighborhood connectivity, topological coefficient and radiality of the PPI network of each protein were calculated using network analyzer (a cytoscape plugin) (Additional file 1: Table S15) [40]. These 9 important topological features were previously used for the identification of Alzheimer Disease associated genes [18].

### Feature selection

We had normalized the features and computed Pearson correlation coefficient (PCC) among the feature pairs using “caret” R package (https://cran.r-project.org/web/packages/caret/index.html). For the basic level of feature selection or filtering, we eliminated the features with high correlation (PCC value > 0.8) with all other features to avoid multicollinearity. We named the normalized and basic level feature selection as normalized and filtered, respectively.

We used ensemble feature selection tool (EFS provided by Neumann et al. [41]) for advanced level feature selection.

### Classification

Identification of disease-associated proteins can be viewed as a binary classification problem with any protein either associated or not associated with the disease. We have employed well-known classifiers like DNN, SVM, NB and RF to distinguish infectious disease and non-disease associated proteins.

#### Deep neural networks (DNN)

**Fig. 4:**
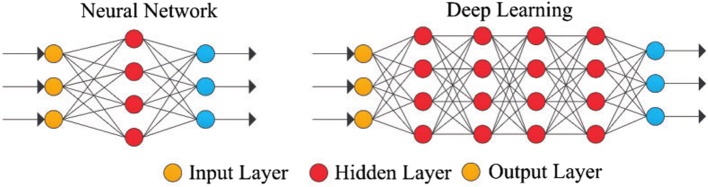
The architecture of simple Deep Neural Networks

Unlike conventional neural networks, modern DNN is more robust and useful for complex classification problems [42]. DNN task can be accomplished by the basic framework of multi-layer neural networks. The simple DNN architecture is shown in Fig. 4. DNN is a hierarchical feature extraction model, usually comprising of multiple level of nonlinearity. This model allows multiple processing layers to learn representation of data with multiple level of abstraction. Because of its performance with diverse problems, DNN is becoming a popular algorithm in the field of computational biology. We have used “h2o” deep learning R package (https://cran.r-project.org/web/packages/h2o/index.html) to predict disease and non-disease associated proteins. We tested hyper-parameter tuning with grid search to optimize the performance measures of the prediction model.

#### Support vector machines (SVM)

SVM is a supervised learning technique for solving binary classification problems [43]. It is a non-probabilistic classification where both the training and testing data are assigned to one group or the other. In addition to linear data, SVM can also handle non-linear data using the kernel trick. We used the “e1071” R package for SVM classification (https://cran.r-project.org/web/packages/e1071/index.html). To find the best performance of the SVM classifier, we tested different combinations of cost and gamma parameters of radial basis function (RBF).

#### Naïve Bayes (NB)

NB is a popular probabilistic classification method based on Bayes theorem [44]. The strong presumption is that the features are independent of each other. We obtained NB classifiers from the Waikato Environment for Knowledge Analysis (WEKA) machine learning toolbox [45].

#### Random Forest (RF)

RF is a learning method based on construction of multiple decision trees [46]. During construction of the trees, randomness was used to create a forest of uncorrelated trees whose prediction ability is higher when working as a committee than the ability of the individual trees. We used WEKA to perform RF classification. Different parameters were tested to find the best performance.

### Performance measures

The performance measures of classification problems such as sensitivity, specificity, accuracy, positive predictive value (PPV), Mathew’s correlation coefficient (MCC) and F1 score were calculated using the similar equations mentioned in our previous study [47]. Here, TP, FP, TN, and FN are defined as below.

#### True positive (TP)

*Infectious disease-associated proteins are correctly identified as infectious disease-associated proteins.*

#### False positive (FP)

*Non-disease associated proteins are incorrectly identified as infectious disease-associated proteins.*

#### True negative (TN)

*Non-disease associated proteins are correctly identified as non-disease associated proteins.*

#### False negative (FN)

*Infectious disease-associated proteins are incorrectly identified as non-disease associated proteins.*

The area under the receiver operating characteristic curve (AUC) was also computed for all cases.

### Functional annotation

The Database for Annotation, Visualization and Integrated Discovery (DAVID) web server was used to identify significant disease ontology and gene ontology enriched terms for highly predicted proteins by the proposed method [48, 49]. We considered only Genetic Association Database (GAD) disease ontology terms with *P*-value < 0.05 [28]. Similarly, we considered only GO biological process terms with *P*-value < 0.05 and false discovery rate (FDR) value < 0.05.

## Supplementary information

**Additional file 1: Table S1.** All the curated infectious diseases-associated human genes from DisGeNET. **Table S2.** All the mapped gene name to uniprot id using mapping table of DisGeNET. **Table S3.** Positive dataset for 10-fold cross-validation. **Table S4.** Positive blind dataset (not used in training or testing of 10-fold cross-validation techniques for developing the prediction model). **Table S5.** All the disease-associated human reviewed proteins in DisGeNET. **Table S6.** All the reviewed human proteins collected from UniProtKB dated 12/01/2018. **Table S7.** All the reviewed human proteins not associated with any diseases. **Table S8.** Negative dataset for 10-fold cross-validation. **Table S9.** Negative blind dataset (not used in training or testing of 10-fold cross-validation techniques for developing the prediction model). **Table S10.** Independent dataset (Befree text mining genes from DisGeNET associated with infectious diseases). **Table S11.** All human protein-protein interactions (PPIs) from Human Protein Reference Database (HPRD) (Release 9). **Table S12.** All unique human in HPRD (Release 9). **Table S13.** All the mapped human protein-protein interactions (PPIs) in uniprot id format. **Table S14.** All the mapped unique human proteins in uniprot. **Table S15.** 9 topological properties of protein-protein interaction networks using HPRD PPIs dataset. **Table S16.** Features wise performance measures on disease and non-disease associated proteins dataset using deep neural network classifier. **Table S17.** Mixed features based performance on disease and non-disease associated proteins dataset. **Table S18.** 10 selected features for normalized and filtered PAAC and Network properties. **Table S19.** 16 selected features for PAAC and Network properties. **Table S20.** Selected features wise performance measures using different classifier. **Table S21.** Prediction result on independent dataset. **Table S22.** Top 100 proteins (genes) are predicted by our proposed DNN based method. **Table S23.** Significantly enriched disease-ontology terms for top 100 proteins (genes) based on Genetic Association Database (GAD). **Table S24.** Significantly enriched gene-ontology biological process terms for top 100 proteins (genes).

**Additional file 2: Figure S1.** Venn diagram of All reviewed and DisGeNET human proteins.

**Additional file 3: Figure S2.** Venn diagram of positive curated and Befree text mining disease-associated proteins (DisGeNET confident score > greater than 0.002738764).

**Additional file 4: Figure S3.** Venn diagram of highly predicted infectious disease-associated proteins and virus and bacteria targeted interaction of human proteins by PHISTO.

**Additional file 5: Figure S4.** Venn diagram of disease ontology terms.

## Data Availability

Source codes, supplementary information are available at https://github.com/ranjan1010/DAG_BarmanEtal2019

## Abbreviations

AAC: Amino acid composition
AUC: Area under receiver operating characteristic curve
CTD: Comparative Toxicogenomics Database
CTD: Conjoint triad descriptors
DAVID: The Database for Annotation, Visualization and Integrated Discovery
DC: Dipeptide composition
DNN: Deep Neural Networks
EFS: Ensemble features selection
FN: False Negative
FP: False Positive
GAD: Genetic Association Database
GO: Gene ontology
GWAS: Genome-wide association study
HPRD: Human Protein Reference Database
LHGDN: Literature Human Gene Derived Network
MAMPs: Microbe-associated molecular patterns
MGD: Mouse Genome Database
MLT: Machine learning techniques
NB: Naïve Bayes
PAAC: Pseudo-amino acid composition
PAMPs: Pathogen-associated molecular patterns
PCC: Pearson correlation coefficient
PPIs: Protein-protein interactions
RF: Random Forest (RF)
RGD: Rat Genome Database
ROC: Receiver operating characteristic curve
SNPs: Single nucleotide polymorphisms
SVM: Support Vector Machine
TN: True Negative
TP: True Positive
WHO: World Health Organization
