# Supplementary figures and images for: Identification of infectious disease-associated host genes using machine learning techniques
Source: BMC Bioinformatics. 2019 Dec 27;20:736. doi: 10.1186/s12859-019-3317-0 (PMC6935192; doi:10.1186/s12859-019-3317-0)

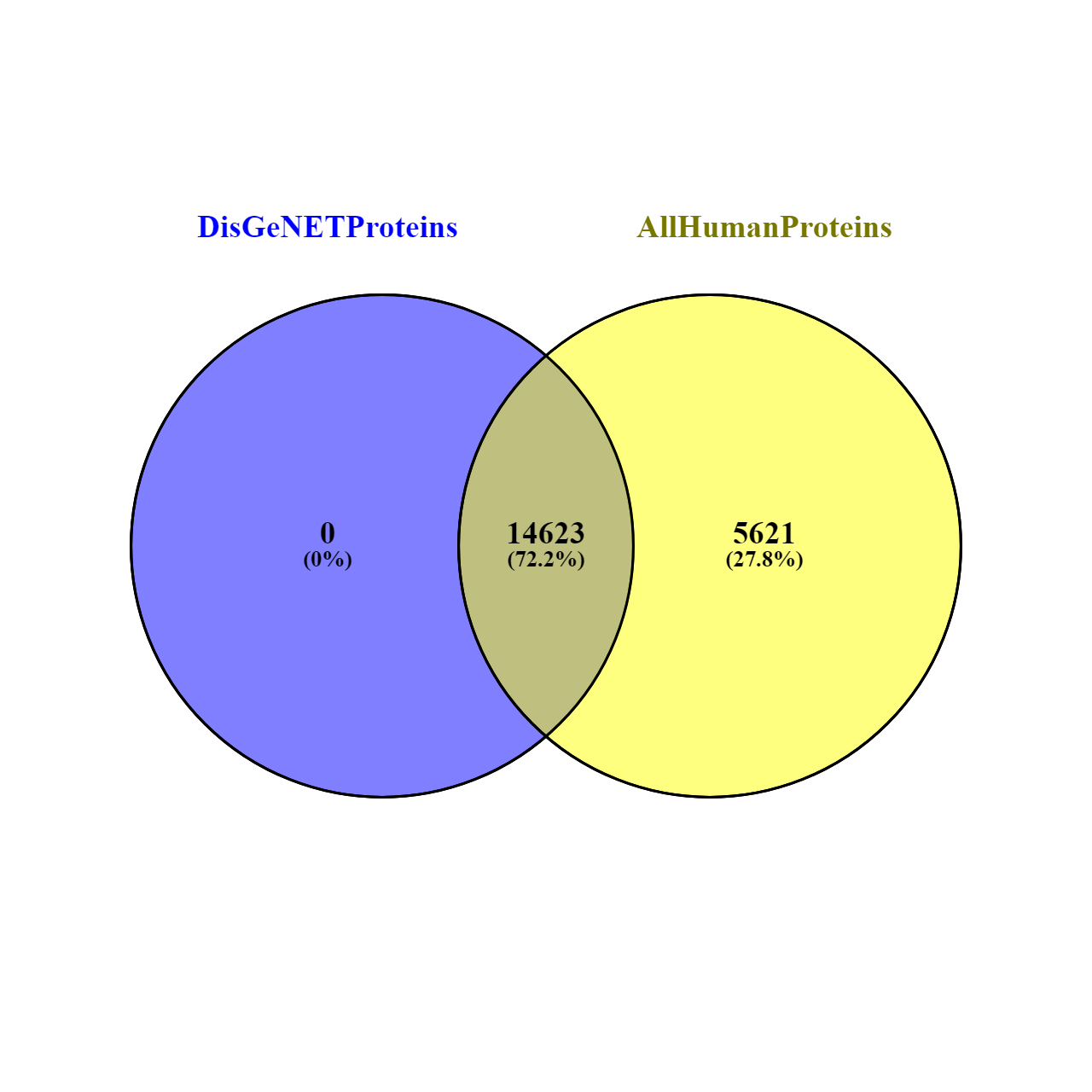

Supplement: Supplementary file 2 — Additional file 2: Figure S1. Venn diagram of All reviewed and DisGeNET human proteins. [file 12859_2019_3317_MOESM2_ESM.tif]

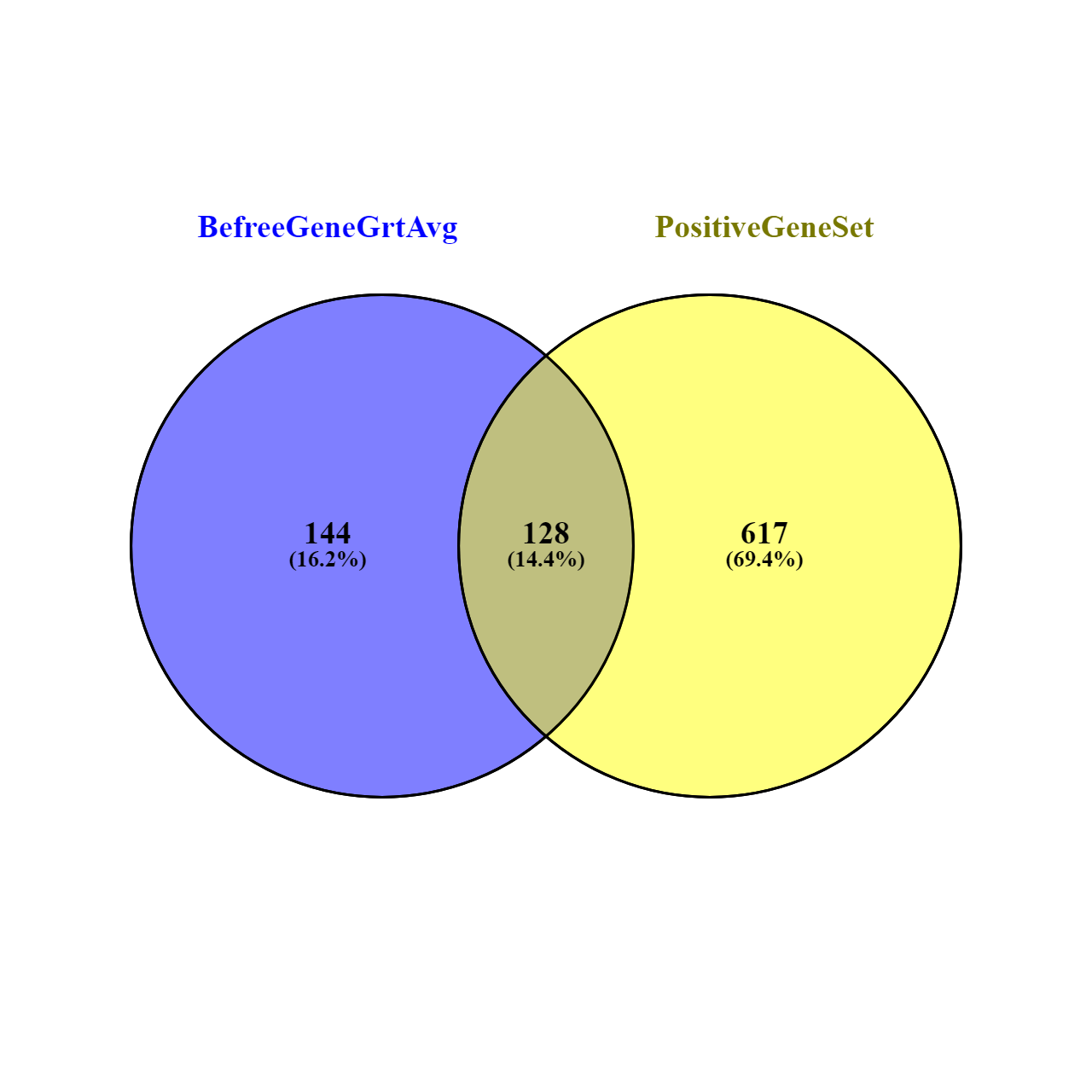

Supplement: Supplementary file 3 — Additional file 3: Figure S2. Venn diagram of positive curated and Befree text mining disease-associated proteins (DisGeNET confident score > greater than 0.002738764). [file 12859_2019_3317_MOESM3_ESM.tif]

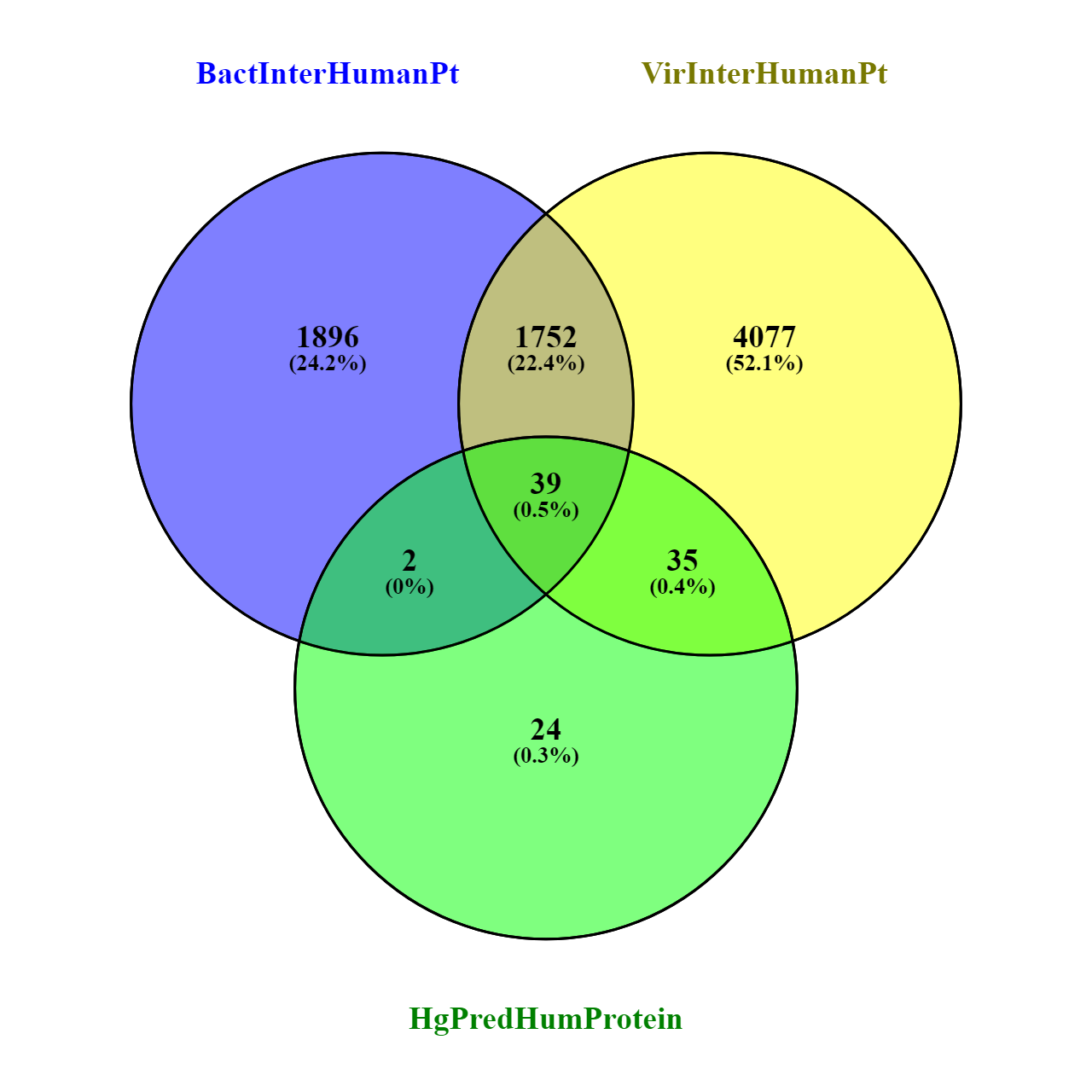

Supplement: Supplementary file 4 — Additional file 4: Figure S3. Venn diagram of highly predicted infectious disease-associated proteins and virus and bacteria targeted interaction of human proteins by PHISTO. [file 12859_2019_3317_MOESM4_ESM.tif]

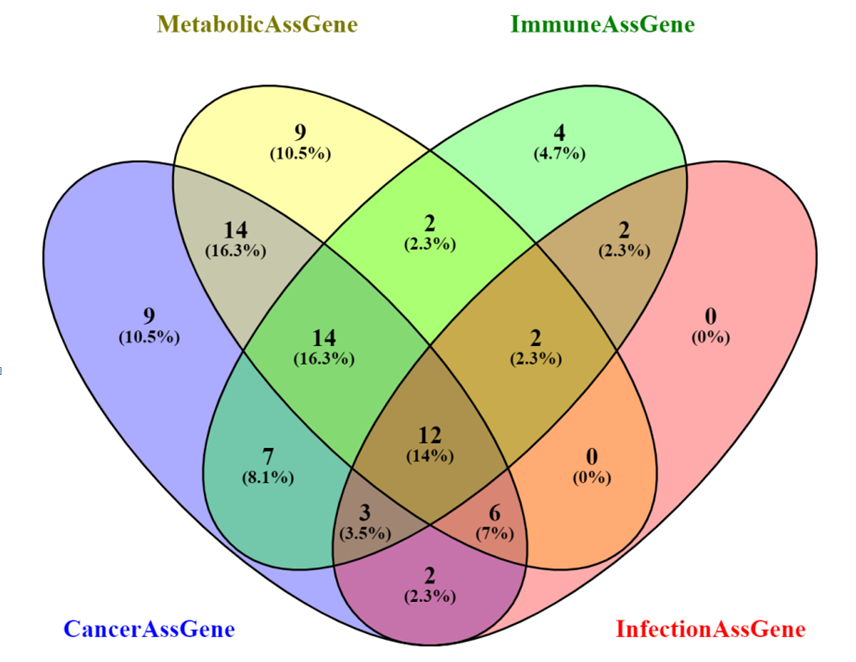

Supplement: Supplementary file 5 — Additional file 5: Figure S4. Venn diagram of disease ontology terms. [file 12859_2019_3317_MOESM5_ESM.tif]
